# The Antioxidant *Cistanche deserticola* Polysaccharide Modulates Gut Microbiota and Redox Homeostasis to Alleviate BAPN-Induced Aortic Dissection in Mice

**DOI:** 10.3390/antiox15070831

**Published:** 2026-06-30

**Authors:** Zhixi Wei, Xinyu Luo, Yi Xia, Mingyang Cui, Peng An, Junjie Luo, Yongting Luo

**Affiliations:** Department of Nutrition and Health, China Agricultural University, Beijing 100193, China; wzx10001000@cau.edu.cn (Z.W.); b202533115881@cau.edu.cn (X.L.); xy1999100845689@163.com (Y.X.); cuimy_86@163.com (M.C.); anpeng@cau.edu.cn (P.A.)

**Keywords:** *Cistanche* polysaccharides, aortic dissection, antioxidant, anti-inflammatory, gut microbiota

## Abstract

Aortic dissection (AD) is a life-threatening vascular disease characterized by progressive vascular remodeling, oxidative stress, and inflammation. Among them, severe oxidative stress and systemic inflammation are important driving factors causing vascular integrity damage. *Cistanche deserticola* polysaccharides (CTPs) have definite deserticola anti-inflammatory and antioxidant properties. However, their influence on the progression of AD remains to be studied. In this study, we investigated the protective effects of CTP in a BAPN-induced mouse model of aortic dissection and explored the underlying mechanisms. CTP administration significantly attenuated aortic dilation and reduced the incidence of aortic dissection, accompanied by suppression of oxidative stress and inflammatory responses, preservation of extracellular matrix integrity, and maintenance of the contractile phenotype of vascular smooth muscle cells. Most importantly, CTP inhibits oxidative stress responses, as evidenced by the recovery of endogenous antioxidant enzyme activity and the reduction in lipid peroxidation. At the same time, CTP also suppresses systemic inflammatory responses. In addition, CTP markedly reshaped gut microbiota composition, characterized by enrichment of *Akkermansia* and *Lachnospiraceae* and reduction in *Desulfovibrio* and *Escherichia-Shigella*. Correlation analyses revealed close associations between gut microbial alterations and antioxidant, vascular remodeling, and smooth muscle cell phenotypic modulation. Collectively, these findings suggest that CTP confers vascular protection against aortic dissection through coordinated regulation of oxidative stress, inflammation, and vascular remodeling. The observed changes in gut microbiota composition may represent an additional mechanism associated with the beneficial effects of CTP and warrant further investigation.

## 1. Introduction

Aortic dissection (AD) is an acute cardiovascular disease with an extremely high mortality rate, characterized by the tearing of the aortic intima, which causes the aortic wall to be divided into a true lumen and a false lumen [1]. An increasing number of studies have confirmed that the inflammatory response plays a key role in the pathogenesis of aortic dissection. Inflammatory cells infiltrating the aortic media release proteases, reactive oxygen species, and pro-inflammatory cytokines, which may lead to apoptosis of smooth muscle cells and degradation of extracellular matrix, ultimately weakening the aortic wall and increasing the probability of dissection [2]. Elevated inflammatory markers, such as IL-6 and TNF-a, are positively correlated with the severity of the disease in patients with aortic dissection, indicating that the synergistic interplay between inflammation and redox imbalance plays a crucial role in the progression of AD [3].

Oxidative stress, closely intertwined with chronic inflammation, has also been increasingly confirmed to be a key link between cardiovascular diseases and intestinal flora imbalance. The trillions of microorganisms residing in the gastrointestinal tract influence host immunity and metabolic homeostasis, and alterations in gut microbial composition and derived metabolites can modulate systemic inflammatory responses and oxidative stress [4]. Gut microbiota-associated metabolites such as short-chain fatty acids (SCFAs), trimethylamine-N-oxide (TMAO), and lipopolysaccharide (LPS) can affect vascular function, immune cell differentiation, and oxidative stress signaling pathways that are relevant to cardiovascular pathology. The role of gut microbiota in atherosclerosis and hypertension has been widely explored. Meanwhile, some studies have demonstrated that microbial imbalance and the inflammatory response fluid it triggers may promote the pathological processes of aortic-related diseases [5].

*Cistanche* polysaccharides (CTPs) are the main active components extracted from *Cistanche*, a traditional herb widely present in East Asia. Existing studies have shown that CTP has antioxidant properties, can regulate the production of cytokines, as well as inflammatory responses and immune cell functions [6]. Notably, CTPs have been shown to modulate gut microbiota composition and microbial metabolite profiles, suggesting a critical role in controlling systemic inflammation via the gut–immune axis. Given that oxidative stress, inflammation, and immune dysregulation are central to the initiation and progression of AD, and that the gut microbiota critically shapes host inflammatory responses, *Cistanche* polysaccharides may represent a novel preventive strategy against AD by targeting gut-mediated antioxidant function. Therefore, CTP may be able to prevent AD through reducing gut-mediated inflammation.

In this study, we employed a β-aminopropionitrile (BAPN)–induced mouse model of aortic dissection to investigate the potential preventive effects of CTP. CTP was administered concomitantly with BAPN induction, and its effects on AD incidence and aortic structural integrity were systematically evaluated. Comprehensive analyses were performed at histopathological and gene expression levels, together with gut microbiota profiling, to elucidate the protective role of CTP against AD and to explore its underlying antioxidant and gut-mediated mechanisms.

## 2. Materials and Methods

### 2.1. Animals and Reagents

Three-week-old male C57/6J mice were purchased from Beijing Vital River Laboratory Animal Technology Limited Liability Company (Beijing, China). All mice were housed in the pathogen-free facility (temperature: 20–26 °C; humidity: 40–70%; pressure: 45 Pa; animal illumination: 15–20 lux; light: 12 h/12 h light/dark cycle). Every cage maintained 6 mice. All mice were maintained under specific pathogen-free conditions with controlled environmental parameters (temperature, 20–26 °C; relative humidity, 40–70%; pressure, 45 Pa; illumination, 15–20 lux) and a 12 h light/dark cycle. Animals were housed at a density of six mice per cage. All experimental procedures were conducted in accordance with the Guiding Principles for the Care and Use of Laboratory Animals and were approved by the Animal Ethics Committee of China Agricultural University (Approval No. AW72406202-5-01, Approval Date: 10 October 2025). CTPs (purity ≥ 98% by the phenol–sulfuric acid method) were obtained from Beijing Kangruina Biotechnology Co., Ltd. (Beijing, China, Cat: LA3617).

### 2.2. Aortic Dissection Model Induction and Treatment

Thirty-six 3-week-old male C57/6J mice were randomly divided into the control group, the BAPN group, and the CTP group. During the induction stage of aortic dissection, the control group of mice was fed with normal feed and water (n = 12). The BAPN group and the CTP group were fed a diet containing 0.4% BAPN. Based on previous research, the CTP group was given CTP (dissolved in distilled water) at a dose of 200 mg/kg/d by gavage [7,8]. All the mice were sacrificed at 25 days. A total of 12 mice were initially included in each experimental group. For downstream analyses, 6 biological replicates per group were used for histological evaluation, biochemical assays, qPCR analyses, and gut microbiota sequencing. Western blot analyses were performed using 3 independent biological replicates per group.

### 2.3. Ultrasound Imaging

Transthoracic echocardiography was performed to evaluate the maximal diameter of the ascending aorta in mice using the Vevo 2100 High-Resolution Imaging System (FUJIFILM VisualSonics, Toronto, ON, Canada) equipped with an 18–38 MHz scanning head (MS400, mouse cardiovascular). Mice were anesthetized with isoflurane (1–2% in oxygen) and maintained in a supine position on a temperature-controlled platform. Two-dimensional (B-mode) and M-mode images were acquired to visualize the ascending aorta and aortic arch. Aortic diameter was measured at end-diastole as an indicator of aortic dissection severity. All measurements were averaged from three consecutive cardiac cycles and analyzed by an investigator blinded to the experimental groups [9].

### 2.4. Histological Analyses

After euthanasia, aortic tissues were carefully harvested, fixed in 4% paraformaldehyde, dehydrated through a graded ethanol series, and embedded in paraffin. Serial transverse sections (5 μm thickness) were prepared for histological evaluation. Masson’s trichrome staining was performed to assess collagen deposition and overall tissue architecture. Elastic fiber integrity and medial structure were examined using Elastic van Gieson (EVG) staining. Alcian blue staining was applied to evaluate acidic mucopolysaccharide and proteoglycan content within the aortic wall. Images were acquired using a Leica DM6B microscope (Leica Microsystems GmbH, Wetzlar, Germany) equipped with 20× or 40× HC PLAN objectives and Leica Application Suite software (version 4.10). Image processing for presentation was performed using Adobe Photoshop and Illustrator (Adobe Systems, San Jose, CA, USA) [10].

### 2.5. Biochemical Assays

Oxidative stress-related parameters were measured in aortic tissue homogenates using commercial assay kits according to the manufacturers’ instructions. Total superoxide dismutase (T-SOD; cat. G4306-48T), malondialdehyde (MDA; cat. G4302-48T), catalase (CAT; cat. G4307-48T), and glutathione peroxidase (GSH-Px; cat. G4310-48T) assay kits and reactive oxygen species (ROS; cat. G1746-100T) were purchased from Wuhan Saiwei Biotechnology Co., Ltd. (Wuhan, China). T-SOD activity was determined based on its ability to inhibit superoxide anion-mediated reactions and expressed as U/mg protein. MDA content, an indicator of lipid peroxidation, was measured using the thiobarbituric acid (TBA) method and expressed as nmol/mg protein. CAT activity was determined by measuring the decomposition rate of hydrogen peroxide and expressed as U/mg protein. GSH-Px activity was measured based on its ability to catalyze the reduction in peroxides and was expressed as U/mg protein. ROS levels were expressed as relative fluorescence units (RFU)/mg protein [11]. According to the manufacturer’s instructions (Wuhan Saiwei Biotechnology Co., Ltd., Wuhan, China), the absorbances at 532, 450, 405, 412, and 520 nm were measured using a microplate reader (Thermo Fisher Scientific, Waltham, MA, USA). The original SOD, CAT, and GSH-Px activities were expressed as U/mg protein, whereas MDA content was expressed as nmol/mg protein. For graphical presentation, all values were normalized to the control group and are presented as relative values.

### 2.6. Real-Time PCR

Total RNA was extracted from aortic tissues using TRIzol reagent [12]. RNA concentration and purity were determined by NanoDrop OneC (Thermo Fisher Scientific, USA), and samples with acceptable purity were used for subsequent experiments. Complementary DNA (cDNA) was synthesized from total RNA using a reverse transcription kit following the manufacturer’s protocol. Quantitative real-time PCR was performed using a SYBR Green PCR Master Mix on a QuantStudio 5 384 Well Block (Thermo Fisher Scientific, USA). Gene expression levels were normalized to the internal reference gene β-actin and calculated using the 2^−ΔΔCt^ method [13]. Primer sequences used for RT-qPCR are listed in Supplementary Table S1.

### 2.7. Western Blotting (WB)

Aortic tissues were homogenized in RIPA lysis buffer supplemented with protease and phosphatase inhibitors [14]. The lysates were centrifuged at 12,000× *g* for 15 min at 4 °C, and the supernatants were collected for protein analysis. Equal amounts of protein were separated by SDS–PAGE and transferred onto polyvinylidene difluoride (PVDF) membranes [15]. Membranes were blocked with 5% non-fat milk in Tris-buffered saline containing 0.1% Tween-20 (TBST) and subsequently incubated overnight at 4 °C with primary antibodies. After washing with TBST, membranes were incubated with appropriate horseradish peroxidase–conjugated secondary antibodies at room temperature. Protein bands were visualized using an enhanced chemiluminescence (ECL) detection system and quantified by densitometric analysis. Target protein expression levels were normalized to β-actin as loading controls.

### 2.8. Gut Microbiota Analysis

Fresh fecal samples were collected under sterile conditions and immediately stored at −80 °C until analysis. Bacterial genomic DNA extraction, 16S rRNA gene amplification, PCR product quality assessment, and sequencing library construction were conducted in accordance with the standard protocols of Majorbio Bio-Pharm Technology Co., Ltd. (Shanghai, China). The V3–V4 hypervariable regions of the bacterial 16S rRNA gene were amplified using universal primers [16]. The QIIME (Quantitative Insights Into Microbial Ecology) software package (version 1.2.8) was used for the following analyses. The operational classification unit (OTU) was tasked with gathering high-quality reads to perform several analyses.

Linear discriminant analysis effect size (LEfSe) was applied to identify differentially abundant taxa across all taxonomic levels between the control and CTP groups [17]. Alpha diversity was evaluated using the ACE and Shannon indices, while beta diversity was assessed by principal component analysis (PCA). Permutational multivariate analysis of variance (PERMANOVA, adonis) was used to determine differences in microbial community structure between groups. The composition and relative abundance of gut microbiota were further analyzed at the phylum and genus levels. Spearman correlation heatmap analysis was performed to assess the associations between gut microbial taxa and serum lipid parameters as well as pathological indicators of atherosclerosis. In addition, functional profiles of the gut microbiota were predicted using Phylogenetic Investigation of Communities by Reconstruction of Unobserved States (PICRUSt) based on comparison with the Kyoto Encyclopedia of Genes and Genomes (KEGG) database [18].

### 2.9. Statistical Analysis

Quantitative analysis of the collected images was performed using ImageJ software (version 1.8.0; National Institutes of Health, Bethesda, MD, USA). Experimental data were statistically analyzed and graphically visualized using OriginPro software 2026 (OriginLab Corporation, Northampton, MA, USA). Data are presented as mean ± standard deviation (SD). Comparisons among multiple groups were performed using one-way analysis of variance (ANOVA) followed by Sidak’s multiple comparisons test. For gut microbiota analyses, alpha diversity indices were compared using the Kruskal–Wallis test, and differentially abundant taxa were identified using LEfSe analysis (version 1.1.01; The Huttenhower Lab, Harvard T.H. Chan School of Public Health, Boston, MA, USA). A *p* value < 0.05 was considered statistically significant.

## 3. Results

### 3.1. CTPs Prevents the Occurrence of AD

**Figure 1:**
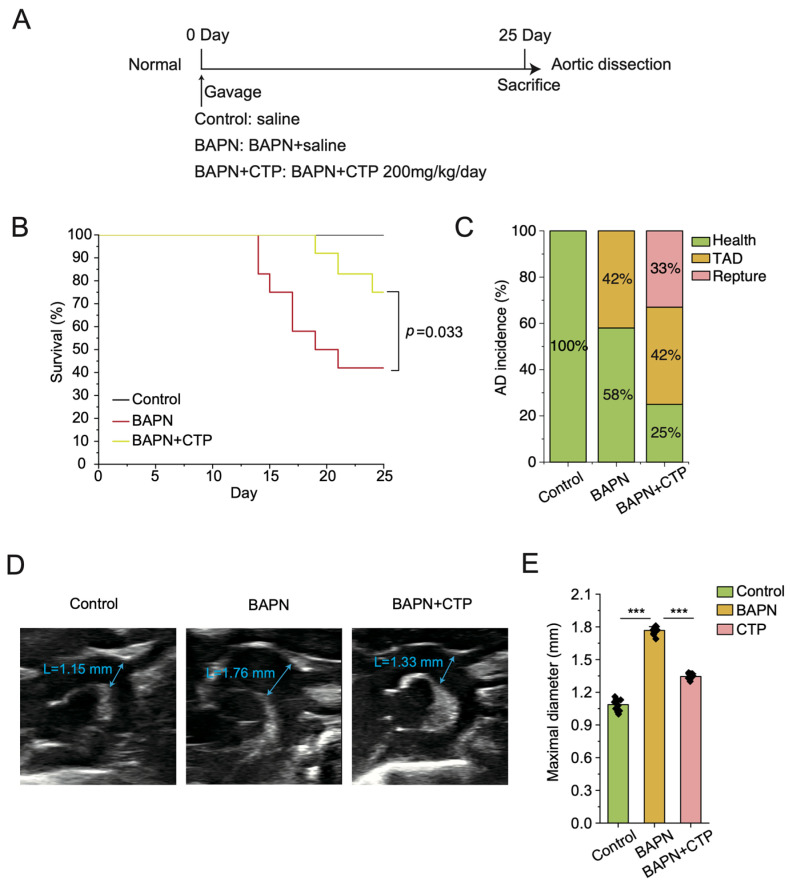
CTP attenuates β-aminopropionitrile-induced aortic dissection in mice. (**A**) A schematic diagram illustrating the timeline for CTP administration in β-aminopropionitrile-induced aortic dissection in mice. (**B**) Survival curves of β-aminopropionitrile-treated/untreated mice with or without CTP treatment at indicated time points. Survival rate was estimated by the Kaplan–Meier method and compared by the log-rank test (n = 12 mice per group). (**C**) The incidence of aortic dissection among β-aminopropionitrile-treated/untreated mice with or without CTP treatment (n = 12 mice per group). (**D**) Representative ultrasound images showing the maximal diameter of thoracic aorta. (**E**) Quantification of maximal diameter for images in (**D**). Each dot represents the maximal diameter of one mouse (n = 12 mice per group). *** *p* < 0.001.

A β-aminopropionitrile (BAPN)–induced mouse model was established to evaluate the preventive effect of Cistanche polysaccharides (CTPs) on aortic dissection (AD) (Figure 1A). Kaplan–Meier survival analysis [6] showed that BAPN administration markedly reduced survival compared with the control group, whereas CTP treatment significantly improved survival in BAPN-challenged mice (Figure 1B).

Consistently, the incidence of AD was substantially increased in the BAPN group, with the majority of mice developing aortic dissection, while CTP administration markedly reduced the occurrence of AD (Figure 1C). Echocardiographic assessment revealed pronounced aortic dilation and structural abnormalities in BAPN-treated mice, which were notably attenuated by CTP treatment (Figure 1D). The measurement of the aortic arch diameter in mice further confirmed that compared with the control group, the BAPN group had severe aortic dilation, while CTP intervention significantly inhibited aortic dilation (Figure 1E).

Collectively, these results demonstrate that CTP effectively prevents the occurrence of aortic dissection and alleviates BAPN-induced aortic structural deterioration in mice.

### 3.2. CTP Attenuates BAPN-Induced Aortic Structural Remodeling

**Figure 2:**
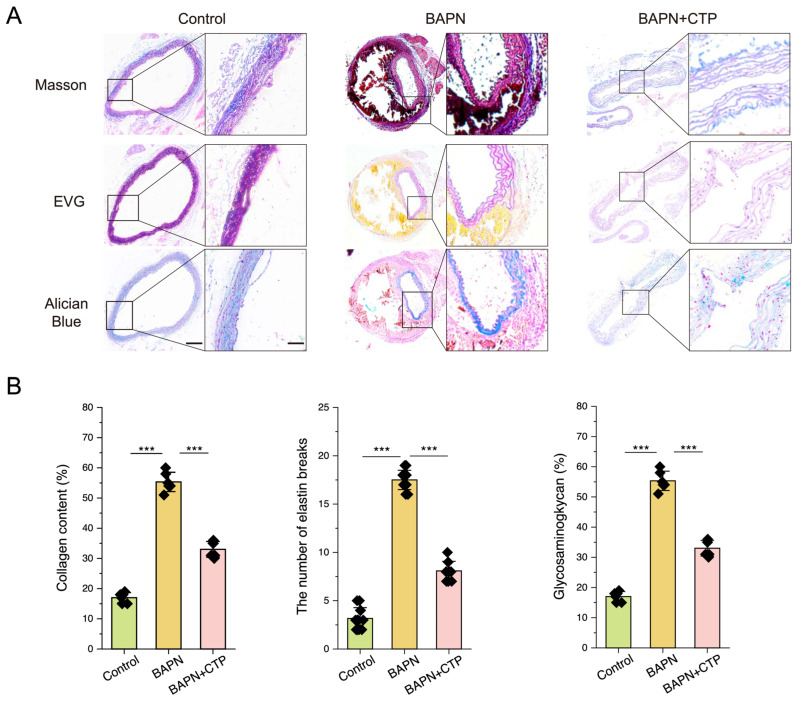
CTP supplementation can reduce BAPN-induced aortic dissection in mice. (**A**) Representative images showing Masson’s trichrome (Masson) (**top**), Elastic van Gieson (**middle**), and Alcian blue (**bottom**) staining in the thoracic ascending aorta from the indicated groups. Arrowheads indicate elastin breaks. Scale bars: 50 μm (low magnification) and 200 μm (high magnification). (**B**) Bar plots showing the collagen content, the number of elastin breaks, and proteoglycan content in thoracic aorta (n = 3 mice per group, two sections per mouse). *** *p* < 0.001.

Histological analyses were conducted to assess the effects of CTP on BAPN-induced aortic pathological remodeling. Masson’s trichrome staining revealed that BAPN administration caused pronounced aortic dilation accompanied by severe disruption of collagen organization and increased fibrotic deposition compared with the control group. In contrast, CTP treatment significantly alleviated these structural abnormalities, with a reduction in collagen deposition and glycosaminoglycan accumulation (Figure 2A,B).

Consistently, Elastic van Gieson (EVG) staining demonstrated extensive elastic fiber fragmentation and disorganization in the aortas of BAPN-treated mice [2], whereas CTP intervention substantially preserved elastic fiber integrity and continuity (Figure 2A,C). Moreover, Alcian blue staining showed excessive accumulation of acidic mucopolysaccharides in the BAPN group [10], indicative of extracellular matrix remodeling, which was significantly attenuated following CTP treatment (Figure 2A,D). Collectively, these findings indicate that CTP effectively mitigates BAPN-induced aortic dilation by preserving extracellular matrix architecture and elastic fiber structure.

### 3.3. CTP Inhibits Oxidative Stress Damage

**Figure 3:**
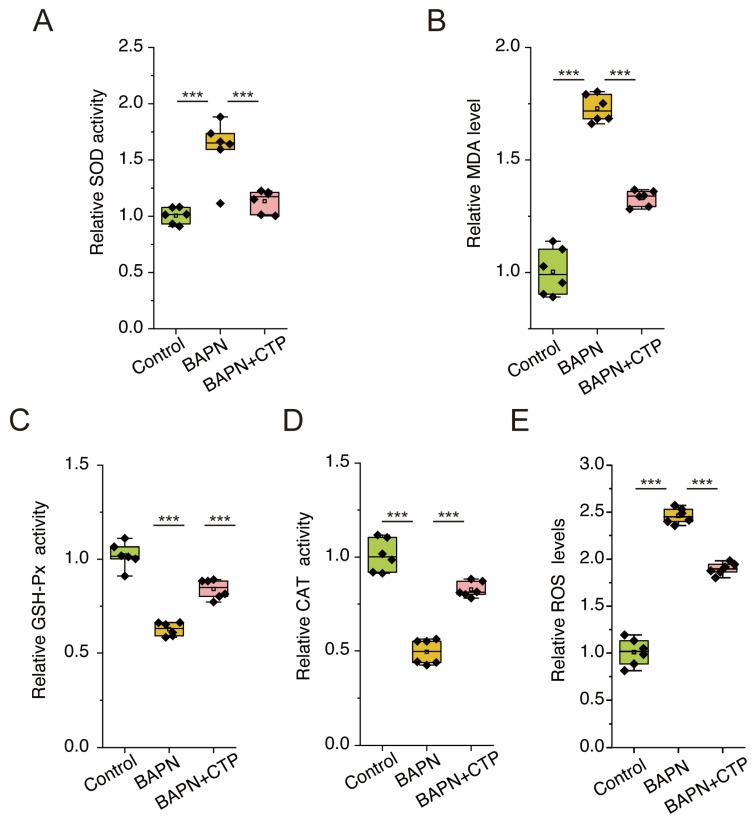
Effects of CTP on oxidative stress in BAPN-induced aortic dissection mice. (**A**) Relative SOD activity, (**B**) relative MDA level, (**C**) relative GSH-Px activity, (**D**) relative CAT activity, and (**E**) relative ROS levels in aortic tissues of control, BAPN, and BAPN + CTP groups (n = 3 mice per group, two technical repetitions per mouse). Data are presented as mean ± SD. *** *p* < 0.001.

An increasing number of studies have shown that oxidative stress injury is closely related to the occurrence of aortic dissection [19]. To investigate whether CTP treatment improved the antioxidant activity of BAPN-induced aortic dissection swelling in mice, oxidative stress indicators (SOD, MDA, GSH-PX, and CAT) in mouse aortic tissues were measured. Compared with the control group, in BAPN-induced oxidative stress of aortic tissue, the activities of antioxidant enzymes such as SOD, GSH-Px, and CAT were significantly decreased, while the level of MDA was significantly increased, and ROS levels were significantly elevated in the BAPN group. It is worth noting that CTP treatment significantly reversed these changes (Figure 3). These results demonstrate that CTP effectively alleviates BAPN-induced oxidative stress by enhancing antioxidant capacity and reducing lipid peroxidation [20].

**Figure 4:**
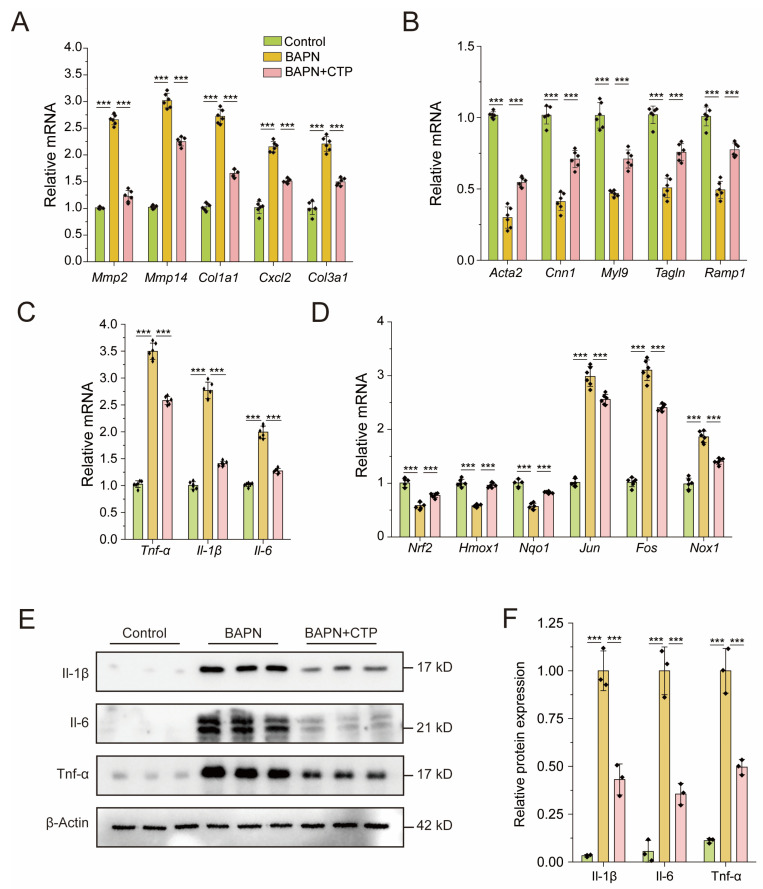
CTP regulates inflammation, oxidative stress, extracellular matrix remodeling, and VSMC phenotypic modulation in BAPN-induced aortic dissection mice. (**A**) Relative mRNA expression levels of extracellular matrix remodeling–related genes (*Mmp2*, *Mmp14*, *Col1a1*, *Cxcl2*, and *Col3a1*). (**B**) Relative mRNA expression levels of inflammatory cytokines (*Tnf-α*, *Il-1β*, and *Il-6*). (**C**) Relative mRNA expression levels of VSMC contractile markers (*Acta2*, *Cnn1*, *Myl9*, *Tagln*, and *Ramp1*). (**D**) Relative mRNA expression levels of antioxidant-related genes (*Nrf2*, *Hmox1*, *Nqo1*, *Jun*, *Fos*, and *Nox1*). Data are presented as mean ± SD (n = 6 per group for mRNA analysis). (**E**) Representative immunoblotting images of contractile and synthetic markers in thoracic aorta tissues from control, BAPN, and BAPN + CTP groups. (**F**) Quantification of the expression levels of selected markers shown in (**E**). Data are presented as mean ± SD (n = 3 per group for western blotting analysis). Statistical significance was determined by one-way analysis of variance (ANOVA) with Sidak’s multiple comparison test. *** *p* < 0.001.

Further, we examined smooth muscle cell contraction, synthesis-related genes, as well as the expression of oxidative stress-related genes. Quantitative RT-qPCR analysis showed that, compared with the control group, the mRNA expression of smooth muscle synthesis-related genes in the aortic tissue of the BAPN group was significantly upregulated, while CTP treatment significantly downregulated the expression levels of these genes (Figure 4A). Consistently, inflammation-related genes exhibit similar expression patterns, with BAPN inducing a significant rise and CTP treatment partially or fully reversing these alterations (Figure 4B). In contrast, BAPN exposure significantly decreased the mRNA expression of the contraction gene of smooth muscle cells in the aorta, while CTP intervention significantly promoted its upregulation (Figure 4C). In addition, the supplementation of CTP can also enhance the expression level of antioxidant factors (*p* < 0.01) (Figure 4D). BAPN also significantly altered the expression of oxidative stress-related genes, including Jun, Fos, and Nox1, whereas CTP treatment markedly reversed these changes. To further validate the inflammatory response at the protein level, Western blot analysis was performed to determine the expression of IL-1β, IL-6, and TNF-α in aortic tissues. Consistent with the qPCR results, the protein expression levels of IL-1β, IL-6, and TNF-α were significantly elevated in the BAPN group compared with the control group, whereas CTP treatment markedly reduced their expression. These findings further confirm the anti-inflammatory effects of CTP in BAPN-induced aortic dissection.

Collectively, these data suggest that CTP exerts a protective effect on BAPN-induced aortic dilation by regulating oxidative stress and inflammation-related signaling pathways as well as the contraction of smooth muscle cells and the expression of synthetic markers [21].

### 3.4. CTP Regulated the Gut Microbiota

**Figure 5:**
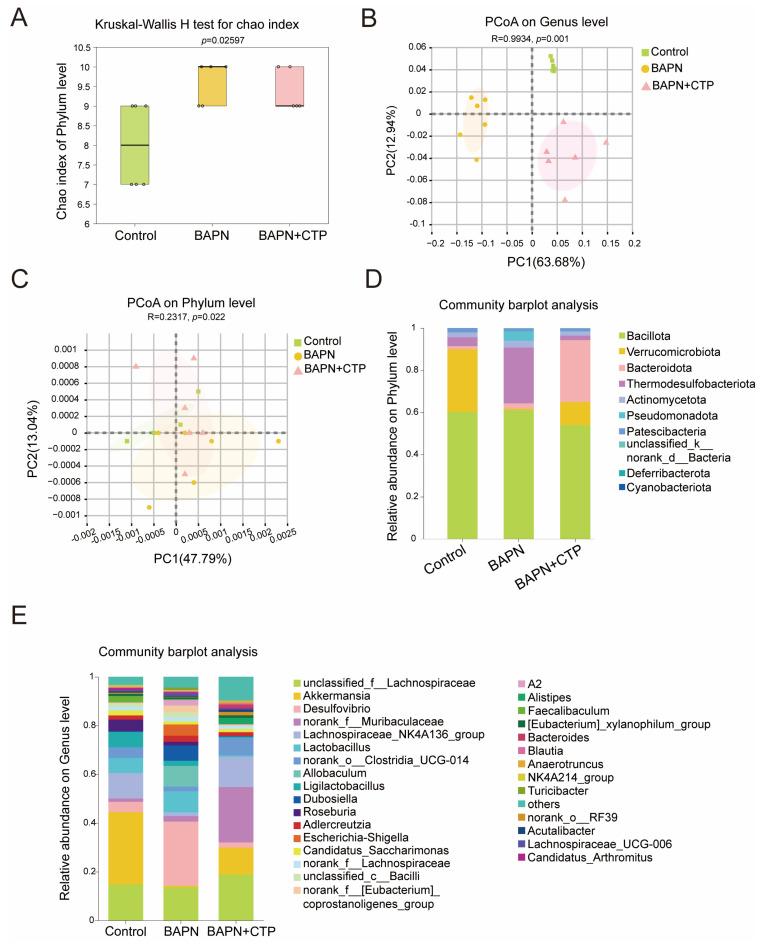
CTP modulates gut microbiota diversity and composition in BAPN-induced aortic dissection mice. (**A**) Alpha diversity analysis based on the Chao index among control, BAPN, and BAPN + CTP groups, assessed using the Kruskal–Wallis H test. (**B**) Principal coordinates analysis (PCoA) of microbial communities at the genus level based on Bray–Curtis distances. (**C**) Principal coordinates analysis (PCoA) at the phylum level. (**D**) Relative abundance of gut microbiota at the phylum level in each group. (**E**) Relative abundance of gut microbiota at the genus level in each group. Data are presented as mean ± SD (n = 6 per group). Statistical significance was determined by the Kruskal–Wallis test for alpha diversity analysis.

The rarefaction curve coverage index saturation approaching 1.0 indicates that the sequencing depth of the gut microbiota is sufficient to replenish the majority of the microbiota diversity in the sample. Furthermore, the rarefaction curves of different groups largely overlapped, indicating that the sequencing depths among the groups were comparable, which ensured the reliability of subsequent microbial diversity and composition analyses (Figure 5A). To evaluate the effects of CTP on gut microbiota dysbiosis induced by BAPN, alpha- and beta-diversity analyses as well as taxonomic profiling were performed. Alpha-diversity analysis at the phylum level was performed using the Chao index to evaluate microbial richness among groups (Figure 5B) [22]. Kruskal–Wallis H test revealed a significant difference in the Chao index among the control, BAPN, and CTP groups. Compared with the control group, BAPN administration significantly altered microbial richness, indicating a marked alteration of gut microbiota composition. Notably, CTP treatment maintained a comparable level of microbial richness to that observed in the BAPN group, suggesting that CTP regulates the composition of the intestinal microbiota rather than microbial richness.

To further evaluate changes in overall microbial community structure, beta-diversity analysis was conducted using principal coordinates analysis (PCoA) [23]. The PCoA plots demonstrated distinct clustering patterns among the three groups, indicating significant differences in gut microbiota composition (Figure 5C,D). Compared with the control group, BAPN induced a pronounced shift in microbial community structure, whereas CTP intervention resulted in a clear separation from the BAPN group and a partial convergence toward the control group. Collectively, these results indicate that CTP modulates gut microbiota diversity and reshapes the microbial community structure disrupted by BAPN.

Community bar plot analysis at the phylum level revealed marked alterations in gut microbiota composition following BAPN administration (Figure 5D). Compared with the control group, BAPN-treated mice exhibited a pronounced increase in Thermodesulfobacteriota accompanied by a reduction in Verrucomicrobiota, indicating significant microbial dysbiosis [24]. Notably, polysaccharide intervention from Cistanche deserticola partially reversed these changes, characterized by a substantial enrichment of Bacteroidota and a marked suppression of Thermodesulfobacteriota. Overall, the microbial community structure in the polysaccharide-treated group shifted toward a profile closer to that of the control group.

Genus-level community analysis revealed pronounced alterations in gut microbiota composition among the three groups (Figure 5E). BAPN treatment resulted in an increased relative abundance of potentially dysbiosis-associated genera, including Desulfovibrio and Escherichia-Shigella, accompanied by a reduction in beneficial commensals such as Akkermansia and Lactobacillus [25]. In contrast, polysaccharide administration markedly reshaped the microbial profile, characterized by enrichment of beneficial genera including Akkermansia, Lachnospiraceae-related taxa, and Muribaculaceae, while suppressing the expansion of BAPN-associated genera.

### 3.5. CTP Affected the Functions of Gut Microbiota

**Figure 6:**
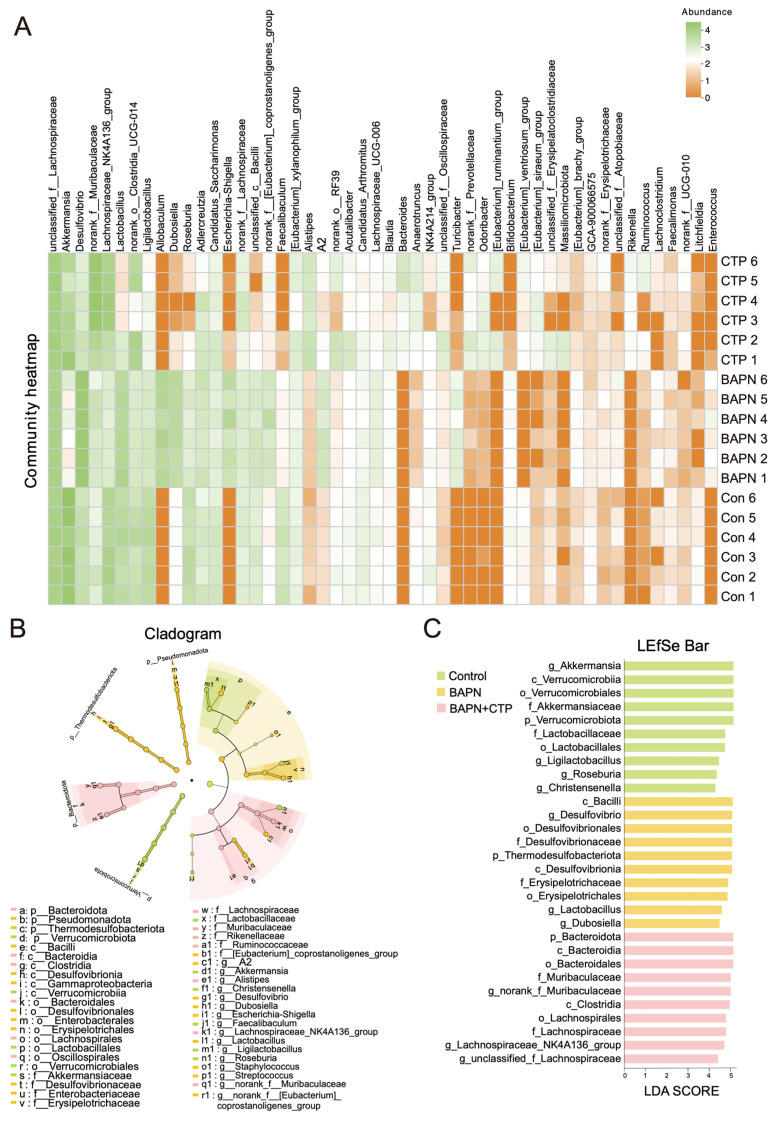
CTP alters gut microbiota composition and identifies key differential taxa in BAPN-induced aortic dissection mice. (**A**) Heatmap of microbial community composition at the genus level, showing relative abundance patterns across control, BAPN, and BAPN + CTP groups. (**B**) Cladogram based on linear discriminant analysis effect size (LEfSe), illustrating taxonomic differences among groups from the phylum to genus level. (**C**) LEfSe analysis showing differentially abundant taxa among groups, with LDA scores indicating the effect size of each feature.

Community heatmap analysis revealed distinct microbial abundance patterns among the control, BAPN, and CTP-treated groups (Figure 6A). Compared with the BAPN group, CTP treatment markedly altered the abundance of multiple genera, characterized by enrichment of Lachnospiraceae and Muribaculaceae-related taxa and a reduction in BAPN-associated bacteria, indicating a pronounced reshaping of gut microbial structure [26]. LEfSe analysis demonstrated clear phylogenetic separation of differentially abundant taxa among the three groups (Figure 6B). Distinct microbial lineages were specifically enriched in the CTP-treated group compared with the BAPN group, suggesting that CTP selectively modulated specific bacterial clades rather than inducing a global microbial shift.

LEfSe bar plot analysis identified multiple microbial taxa with significantly higher LDA scores in the CTP-treated group, indicating CTP-specific microbial biomarkers [27]. In contrast, BAPN treatment was associated with enrichment of a separate set of taxa, highlighting differential microbial signatures between disease induction and polysaccharide intervention. Moreover, Spearman correlation analysis showed that CTP-associated taxa exhibited favorable correlation patterns with host-related indicators, whereas BAPN-enriched bacteria were linked to dysbiosis-associated features (Figure 6C). Collectively, these findings indicate that CTP modulates gut microbiota structure and function, shifting the microbial ecosystem toward a more balanced and potentially protective profile.

### 3.6. Correlation Analysis Between Gut Microbiota and Metabolic Phenotypes

Spearman correlation analysis was performed to evaluate the associations between gut microbiota and vascular pathological indicators, including inflammation, oxidative stress, extracellular matrix remodeling, and vascular smooth muscle cell (VSMC) phenotypic markers [28].

**Figure 7:**
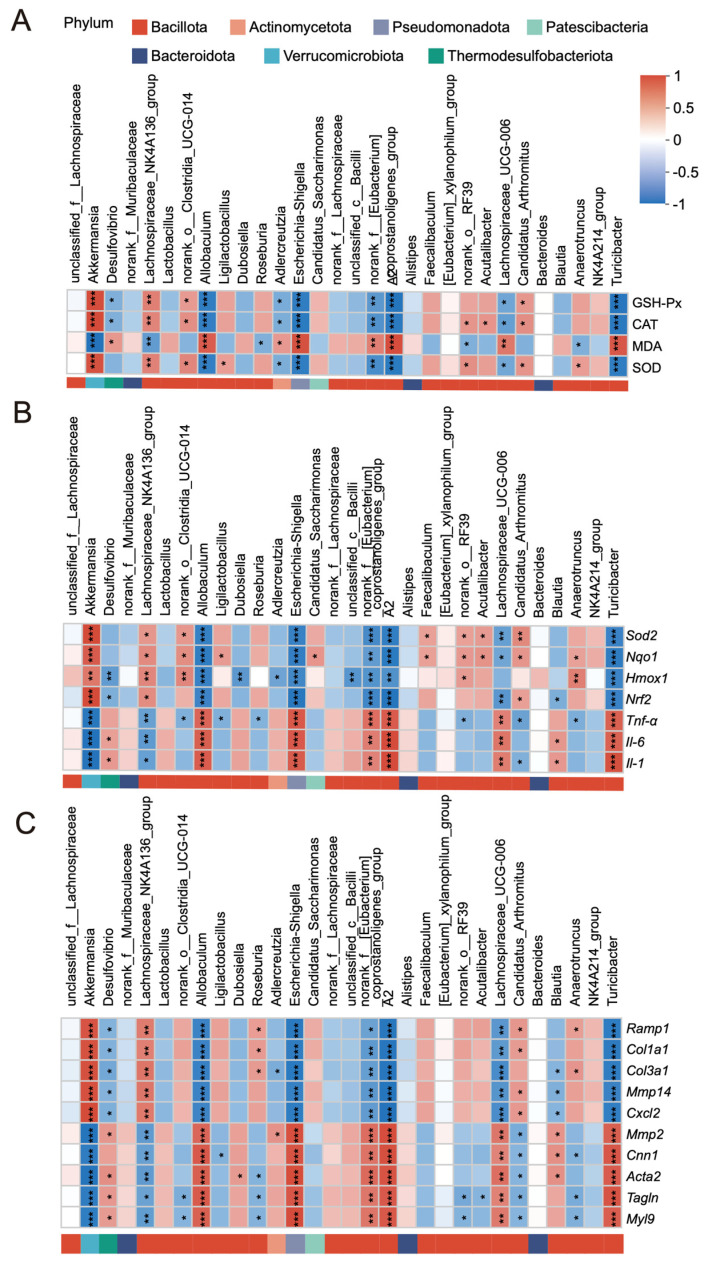
Correlation analysis between gut microbiota and vascular pathological parameters in BAPN-induced aortic dissection mice. (**A**) Spearman correlation heatmap showing associations between gut microbiota and oxidative stress–related biochemical parameters (GSH-Px, CAT, MDA, and SOD). (**B**) Spearman correlation heatmap showing associations between gut microbiota and inflammatory cytokines and antioxidant-related genes (*Il-1β*, *Il-6*, *Tnf-α*, *Nrf2*, *Hmox1*, *Nqo1*, and *Sod2*). (**C**) Spearman correlation heatmap showing associations between gut microbiota (genus level) and vascular smooth muscle cell (VSMC) phenotypic markers and extracellular matrix remodeling–related genes (*Ramp1*, *Col1a1*, *Col3a1*, *Mmp14*, *Cxcl2*, *Mmp2*, *Cnn1*, *Acta2*, *Tagln*, and *Myl9*). Color scale represents Spearman’s correlation coefficient (r), with red indicating positive correlation and blue indicating negative correlation. Statistical significance is indicated as * *p* < 0.05, ** *p* < 0.01, and *** *p* < 0.001.

Beneficial taxa enriched following CTP treatment, such as Akkermansia and members of the Lachnospiraceae family, exhibited significant negative correlations with the lipid peroxidation marker MDA, while showing positive correlations with antioxidant enzyme activities (SOD, CAT, and GSH-Px) (Figure 7A), antioxidant-related genes (Nrf2, Ho-1, Nqo1, and Sod2), pro-inflammatory cytokines (Il-1β, Il-6, and Tnf-α) (Figure 7B), extracellular matrix degradation markers (Mmp2 and Mmp14), and VSMC contractile markers (Acta2, Tagln, and Myl9) (Figure 7C).

In contrast, dysbiosis-associated taxa, including Desulfovibrio and Escherichia-Shigella, displayed the opposite correlation pattern, being positively correlated with inflammatory cytokines, matrix-degrading enzymes, and MDA levels, while negatively correlated with antioxidant gene expression, antioxidant enzyme activities, and VSMC contractile markers [29]. Additionally, several taxa, such as Muribaculaceae and other members of Lachnospiraceae, showed consistent correlation trends across both gene expression and biochemical parameters, further supporting a close association between gut microbiota composition and vascular pathological features [30].

## 4. Discussion

Aortic dissection (AD) is a life-threatening vascular disease characterized by progressive degeneration of the aortic wall, in which oxidative stress, extracellular matrix degradation, and vascular smooth muscle cell dysfunction play critical pathogenic roles [31]. Despite advances in surgical and medical management, effective preventive strategies targeting upstream pathological processes remain limited. *Cistanche deserticola* polysaccharides (CTPs) have been reported to possess antioxidant, anti-inflammatory, and metabolic regulatory activities [32], but their effects on AD have not been investigated. In the present study, CTP significantly reduced the incidence and severity of BAPN-induced AD, attenuated aortic dilation, alleviated oxidative stress and inflammatory responses, and modulated gut microbiota composition. These findings suggest that CTP exerts protective effects against AD and may represent a promising natural bioactive compound for disease prevention.

Oxidative stress is a key contributor to aortic wall degeneration and AD progression. Consistent with previous reports demonstrating the cardiovascular protective effects of natural polysaccharides [33]. BAPN exposure in the present study induced marked oxidative imbalance, characterized by increased MDA levels and decreased activities of SOD, GSH-Px, and CAT. CTP treatment effectively reversed these alterations, restoring antioxidant capacity and redox homeostasis. Furthermore, CTP attenuated pathological remodeling, inflammatory infiltration, and lesion progression, indicating improved vascular stability and structural integrity.

In addition to its vascular protective effects, CTP improved lipid metabolic parameters, as evidenced by reduced TG and LDL-C levels and increased HDL-C levels, consistent with previous reports describing the lipid-lowering and cardioprotective properties of polysaccharides derived from medicinal plants [34]. CTPs also exhibited significant antioxidant and anti-inflammatory activities, which were reflected by improved redox homeostasis and reduced expression of inflammatory cytokines. Western blot analysis further confirmed decreased protein levels of IL-1β, IL-6, and TNF-α following CTP treatment.

Vascular smooth muscle cell (VSMC) phenotypic switching is a hallmark of aortic remodeling and dissection [21]. In the present study, CTP increased the expression of contractile markers while reducing synthetic markers, suggesting preservation of the contractile VSMC phenotype. Because VSMC phenotypic modulation is closely associated with oxidative stress and inflammatory signaling [21], these findings may reflect, at least in part, the ability of CTP to alleviate oxidative and inflammatory injury. Preservation of the contractile phenotype may contribute to maintaining aortic wall integrity and limiting pathological vascular remodeling. The anti-inflammatory effects of CTP were further supported by Western blot analysis, which demonstrated reduced protein expression of IL-1β, IL-6, and TNF-α following CTP treatment, consistent with the observed transcriptional changes.

Gut microbiota dysbiosis has been increasingly associated with vascular inflammation, oxidative stress, and vascular smooth muscle cell (VSMC) dysfunction through microbial metabolites and immune signaling pathways [35]. In the present study, CTP significantly altered gut microbiota composition, enriching beneficial taxa such as *Akkermansia* and *Lachnospiraceae* while reducing dysbiosis-associated bacteria, including *Desulfovibrio* and *Escherichia-Shigella*. Previous studies have shown that *Akkermansia* and *Lachnospiraceae* contribute to gut barrier integrity and suppress inflammatory responses, whereas *Desulfovibrio* and *Escherichia-Shigella* are important sources of endotoxins that promote oxidative stress and inflammation [26]. Consistent with these findings, correlation analyses revealed that beneficial taxa were positively associated with antioxidant-related genes and VSMC contractile markers, whereas dysbiosis-associated taxa were positively correlated with inflammatory cytokines and extracellular matrix degradation markers. These observations suggest that CTP-induced gut microbiota remodeling is closely associated with reduced oxidative stress, attenuated inflammation, preservation of vascular structure, and maintenance of the contractile VSMC phenotype. Although causality remains to be established, the present findings support a potential link between gut microbial alterations and vascular protection in aortic dissection [36].

Spearman correlation analysis revealed significant associations between gut microbiota composition and key pathological processes, including inflammation, oxidative stress, extracellular matrix remodeling, and VSMC phenotypic modulation. Beneficial taxa enriched following CTP treatment, particularly *Akkermansia* and *Lachnospiraceae*, were negatively correlated with pro-inflammatory cytokines, oxidative stress markers, matrix-degrading enzymes, and lipid peroxidation levels, while positively correlated with antioxidant-related genes, antioxidant enzyme activities, and VSMC contractile markers. These findings suggest that enrichment of these taxa is associated with reduced oxidative stress and inflammation, enhanced antioxidant defenses, and preservation of vascular integrity [37]. In contrast, dysbiosis-associated taxa, including *Desulfovibrio* and *Escherichia-Shigella*, showed opposite correlations, being positively associated with inflammatory mediators, oxidative stress markers, and extracellular matrix degradation, and negatively correlated with antioxidant gene expression and VSMC contractile markers. As these taxa have been linked to endotoxin production and inflammatory activation [36], their enrichment may contribute to vascular injury and remodeling. Collectively, these findings suggest that CTP-induced microbial remodeling is associated with a shift toward a less inflammatory and oxidative microenvironment [37,38,39]. Given the limited absorption of intact dietary polysaccharides, direct actions of CTP on vascular cells are likely restricted. Thus, the vascular protective effects of CTP may be associated with microbiota-mediated modulation of oxidative stress and inflammatory responses [33].

## 5. Conclusions

**Figure 8:**
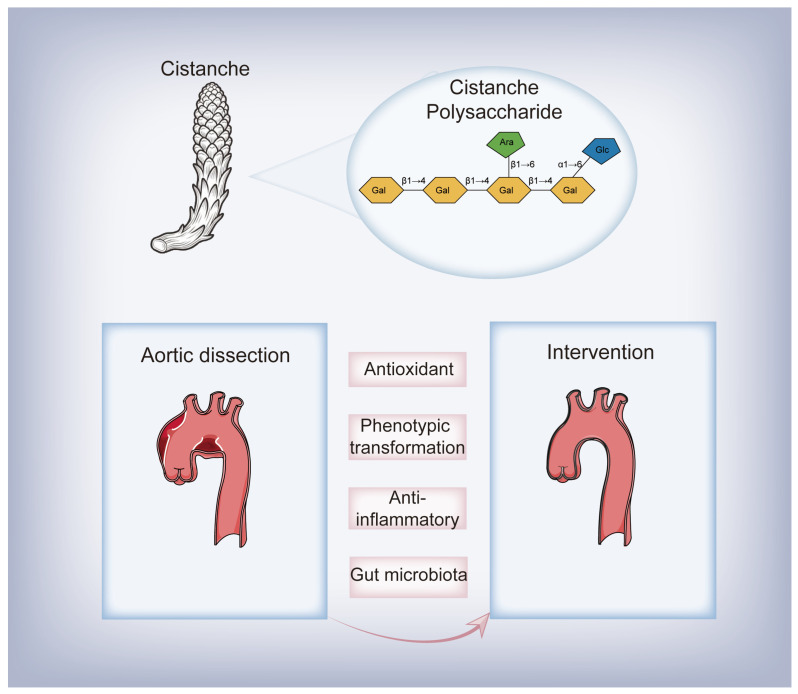
Schematic illustration of the protective effects of CTP against aortic dissection. CTP treatment was associated with reduced oxidative stress and inflammation, preservation of vascular smooth muscle cell phenotype, and alterations in gut microbiota composition. These changes may collectively contribute to vascular protection. This schematic represents a hypothetical model based on the present findings and published evidence and does not imply direct causal relationships among all components.

In summary, CTP effectively attenuates aortic dilation and enhances vascular stability primarily through its profound antioxidant and anti-inflammatory capacities (Figure 8). CTP exerts protective effects against aortic dissection by coordinately modulating oxidative stress, inflammatory responses, vascular smooth muscle cell phenotypic transformation, and gut microbiota composition. These findings highlight a multi-target regulatory mechanism underlying CTP-mediated vascular protection and suggest its potential as a promising therapeutic strategy for aortic dissection.

## Data Availability

Data are contained within the article and Supplementary Materials.

## Supplementary Materials

The following supporting information can be downloaded at https://www.mdpi.com/article/10.3390/antiox15070831/s1. Table S1: Target gene and primer sequences.

## Abbreviations

The following abbreviations are used in this manuscript:

AD	Aortic Dissection
CTP	*Cistanche* polysaccharides
BAPN	β-aminopropionitrile
EVG	Elastic van Gieson
SOD	Superoxide Dismutase
MDA	Malondialdehyde
CAT	Catalase
GSH-PX	Glutathione Peroxidase
