# Supplementary material for: The Antioxidant Cistanche deserticola Polysaccharide Modulates Gut Microbiota and Redox Homeostasis to Alleviate BAPN-Induced Aortic Dissection in Mice
Source: Antioxidants (Basel). 2026 Jun 30;15(7):831. doi: 10.3390/antiox15070831 (PMC13404642; doi:10.3390/antiox15070831)
Supplement: Supplementary file 1 [file antioxidants-15-00831-s001.zip › antioxidants-4344621-supplementary.pdf]

Supplementary Table S1. Target genes and primer sequences

Genes	Accession number	Forward (5′-3′)	Reverse (5′-3′)
Mmp2	NM_008610	GGTGACCTTGACCAGAACACCAT	GGATGTGCCACAAAAGTGCAGG
Mmp14	NC_000080.7	GGATGGACACAGAGAACTTCGTG	CGAGAGGTAGTTCTGGGTTGAG
Col1a1	NC_000077.7	GCTCCTCTTAGGGGCCACT	CCACGTCTCACCATTGGGG
Cxcl2	NC_000071.7	CCAACCACCAGGCTACAGG	GCGTCACACTCAAGCTCTG
Col3a1	NC_000002.12	CTGTAACATGGAAACTGGGGAAA	CCATAGCTGAACTGAAAACCACC
Acta2	NM_001613.2	GTTTTGTGGATCAGCGCCTC	TTCCTGACCACTAGAGGGGG
Cnn1	NM_001299.5	GCAGTGGACACACGCATTTT	AACAACTGGCCCCAAGACTC
Myl9	NM_006097	GCGCCGAGGACTTTTCTTCT	TCTTGGCCTTGGCTCTCTTG
Tagln	NC_000075.7	GCAGATGGAACAGGTGGCTCAA	CCCAAAGCCATTAGAGTCCTCTG
Ramp1	NM_016894.2	GAGACTATTGGGAAGACGCTATG	CTCCTCCAGACCACCAGTG
Tnf-α	NM_013693	GCCGGACTCATCGTACTCC	TTTGCTACGACGTGGGCTAC
Il-1β	NM_008361	TGCCACCTTTTGACAGTGATG	TGCCACCTTTTGACAGTGATG
Il-6	NM_031168	TGCCACCTTTTGACAGTGATG	CGCACTAGGTTTGCCGAGTA
Nrf2	NC_000068.8	TCTTGGAGTAAGTCGAGAAGTGT	GTTGGCAGATCCACTGGTTTT
Hmox1	NC_000074.7	CACTCTGGAGATGACACCTGAG	GTGTTCCTCTGTCAGCATCACC
Nqo1	NC_000074.7	GCCGAACACAAGAAGCTGGAAG	GGCAAATCCTGCTACGAGCACT
β-Actin	NM_007393	GTGACGTTGACATCCGTAAAGA	GCCGGACTCATCGTACTCC
